# A comparison of two personalization and adaptive cognitive rehabilitation approaches: a randomized controlled trial with chronic stroke patients

**DOI:** 10.1186/s12984-020-00691-5

**Published:** 2020-06-16

**Authors:** Ana Lúcia Faria, Maria Salomé Pinho, Sergi Bermúdez i Badia

**Affiliations:** 1 Madeira Interactive Technologies Institute, Universidade da Madeira, Funchal, Portugal; 2 Faculdade de Psicologia e de Ciências da Educação, Universidade de Coimbra, Coimbra, Portugal; 3 NOVA-LINCS, Universidade NOVA de Lisboa, Lisbon, Portugal; 4 Laboratório de Memória, Linguagem e Funções Executivas, Coimbra, Portugal; 5 Centro de Ciências Exatas e da Engenharia, Universidade da Madeira, Funchal, Portugal

**Keywords:** Cognitive rehabilitation, Virtual reality, Stroke, Ecological validity

## Abstract

**Background:**

Paper-and-pencil tasks are still widely used for cognitive rehabilitation despite the proliferation of new computer-based methods, like VR-based simulations of ADL’s. Studies have established construct validity of VR assessment tools with their paper-and-pencil version by demonstrating significant associations with their traditional construct-driven measures. However, VR rehabilitation intervention tools are mostly developed to include mechanisms such as personalization and adaptation, elements that are disregarded in their paper-and-pencil counterparts, which is a strong limitation of comparison studies. Here we compare the clinical impact of a personalized and adapted paper-and-pencil training and a content equivalent and more ecologically valid VR-based ADL’s simulation.

**Methods:**

We have performed a trial with 36 stroke patients comparing Reh@City v2.0 (adaptive cognitive training through everyday tasks VR simulations) with Task Generator (TG: content equivalent and adaptive paper-and-pencil training). The intervention comprised 12 sessions, with a neuropsychological assessment pre, post-intervention and follow-up, having as primary outcomes: general cognitive functioning (assessed by the Montreal Cognitive Assessment - MoCA), attention, memory, executive functions and language specific domains.

**Results:**

A within-group analysis revealed that the Reh@City v2.0 improved general cognitive functioning, attention, visuospatial ability and executive functions. These improvements generalized to verbal memory, processing speed and self-perceived cognitive deficits specific assessments. TG only improved in orientation domain on the MoCA, and specific processing speed and verbal memory outcomes. However, at follow-up, processing speed and verbal memory improvements were maintained, and a new one was revealed in language. A between-groups analysis revealed Reh@City v2.0 superiority in general cognitive functioning, visuospatial ability, and executive functions on the MoCA.

**Conclusions:**

The Reh@City v2.0 intervention with higher ecological validity revealed higher effectiveness with improvements in different cognitive domains and self-perceived cognitive deficits in everyday life, and the TG intervention retained fewer cognitive gains for longer.

**Trial registration:**

The trial is registered at ClinicalTrials.gov, number NCT02857803. Registered 5 August 2016, .

## Background

### Cognitive rehabilitation after stroke

Stroke is a leading cause of long-term acquired disability in adults [1], predisposing patients toward institutionalization and poorer quality of life [2]. Over the coming decades, the incidence of post-stroke disability is expected to increase by 35% due to the rising prevalence of cerebrovascular risk and advances in medicine which are reducing post-stroke mortality rates [3]. Historically, stroke rehabilitation has been focused on motor rehabilitation [4, 5]. However, post-stroke cognitive deficits are pervasive causing disability with major impacts on quality of life and independence on everyday life activities [6, 7]. In the last years, attention to the impact of cognitive deficits has been growing [8] and finding new ways to improve cognition after stroke is considered a priority [9]. Also, more recently, the International Stroke Recovery and Rehabilitation Alliance 2018 working group has identified post-stroke cognitive impairments as a research priority [10].

Regardless of the many new developments in cognitive rehabilitation programs and applications, limited data on the effectiveness of cognitive rehabilitation is available because of the heterogeneity of participants, interventions, and outcome measures [11]. Results from recent reviews corroborate that cognitive rehabilitation has a positive impact on post-stroke cognitive outcomes [12, 13], although of small magnitude (Hedges’ g = 0.48) [12]. This result is in line with the quantitative [14] and qualitative [15–17] findings of previous reviews that have analyzed the effect of cognitive rehabilitation across multiple cognitive domains.

### Is cognitive rehabilitation’s impact small or are we missing better cognitive rehabilitation methodologies?

Paper-and-pencil tasks are still the most widely used methods for cognitive rehabilitation because of their accessibility, ease of use, clinical validity and reduced cost [18]. In the last years, computer-based versions of these traditional tasks are also starting to become clinically accepted [19, 20]. However, there is an absence of specific methodologies that inform health professionals which tasks to apply and under what clinical conditions [21]. Consequently, rehabilitation professionals perform a selection of tasks based on their clinical experience, missing scientific foundations [22]. We have proposed an objective and quantitative framework for the creation of personalized cognitive rehabilitation tasks based on a participatory design strategy with health professionals [23]. In this work, through computational modeling, the authors operationalized 11 paper-and-pencil tasks and developed an Information and Communication Technologies based tool - the Task Generator (TG) - to tailor each of those 11 paper-and-pencil tasks to each patient in the domains of attention, memory, language and executive functions. A clinical evaluation of the TG with twenty stroke patients showed that the TG is able to adapt task parameters and difficulty levels according to patient’s cognitive assessment, and provide a comprehensive cognitive training [24]. However, although it has been shown that rehabilitation strategies based on paper-and-pencil tasks can be personalized and adapted [24, 25], this approach presents a limited transfer to performance in activities of daily living (ADL) [18].

Over the last years, rehabilitation methodologies based on virtual reality (VR) have been developed as promising solutions to improve cognitive functions [26, 27]. VR-based tools have shown potential and to be ideal environments to incorporate cognitive tasks within the simulation of ADL’s [28]. A recent trial with a VR-based simulation of everyday life activities (like going to the pharmacy, buying grocery at the supermarket, paying the water bill) suggested that an ecologically valid intervention has more impact than conventional methods (cognitive training using puzzles, calculus, problem resolution and shape sorting) in cognitive rehabilitation of stroke patients [29]. Also, some of these VR-based systems allow the integration of motor training [30] and recent studies have already shown benefits of performing simultaneous motor and cognitive training with stroke patients using VR [31, 32]. Yet, there is still an insufficient number of rigorous trials to clinically validate VR methods [12] and there are difficulties associated with the limited access which results in a low adoption by health professionals who still prefer mostly use paper-and-pencil interventions [33].

In general, existing ecologically-valid VR-based environments are simulations of cities [29, 34–38], kitchens [39–45], streets [46–51], supermarkets [52–56], malls and other shopping scenarios [57–61]. Of these, only rare cases take into account training personalization according to patient cognitive profile and session-to-session adaptation [29, 36, 38, 41]. Additionally, the results of studies comparing VR cognitive interventions with standard occupational therapy or neuropsychology cognitive paper-and-pencil training are fundamentally subjective as control interventions. OT does not consider cognition as the main training focus, and neuropsychology paper-and-pencil training tasks are too similar to the cognitive assessment scales; additionally, both approaches do not incorporate personalization and dynamic adaptation to performance. Hence, even if rehabilitation sessions last the same, these interventions are not equivalent as they are delivered with uncontrolled difficulty levels and cognitive demands. Personalized rehabilitation is defined as involving an assessment of each patient’s impairments and performing a tailored intervention to his cognitive profile in the different domains. Instead, adaptation deals with the dynamic adjustment of the tasks’ cognitive demands according to the patients’ performance along the intervention sessions, therefore avoiding boredom (tasks that are to easy to solve) or frustration (tasks that are too difficult to solve).

Here we try to address some of the existing limitations in the validation of VR-based cognitive rehabilitation tools. In this study we compared two task content equivalent rehabilitation tools developed under the same personalization and adaptation framework [23]: the TG and the Reh@City v2.0. This framework allows us to make sure that both tools deliver the same controlled adaptation and personalization of difficulty levels, and address the same cognitive demands. Hence, this comparison allows identifying the specific impact of increasing ecological validity of training through VR simulations of ADLs over the same training delivered through clinically accepted paper-and-pencil equivalent tasks. These findings will further inform on the specific benefits of ecologically valid environments delivered though VR and encourage the adoption of these technologies by health professionals.

## Methods

### Participants and trial design

Participants were selected based on the following inclusion criteria: no more than 75 years old; first stroke episode and at least at 6 months post-stroke (chronic phase); no hemi-spatial neglect as assessed by the clinicians with the Line Bisection test [62]; capacity to be seated; minimum of 2 years of schooling (since in Portugal there are quite high rates of illiteracy in the elder populations) and motivation to participate in the study. Patients with a total score of more than two standard deviations below the mean score for age and education in the Montreal Cognitive Assessment (MoCA) [63, 64] were excluded to ensure uniformity and enough cognitive capacity to participate in the rehabilitation interventions. Patients with severe depressive symptomatology, as assessed by the Beck Depression Inventory II [65, 66], were also excluded because its impact on cognitive functioning. Additionally, patients could not have been undergoing OT at least 2 months before the study. The study was previously approved by the Madeira Health Service Ethical Committee (reference number: 13/2016), and all the patients gave informed consent previous to participation.

**Fig. 1:**
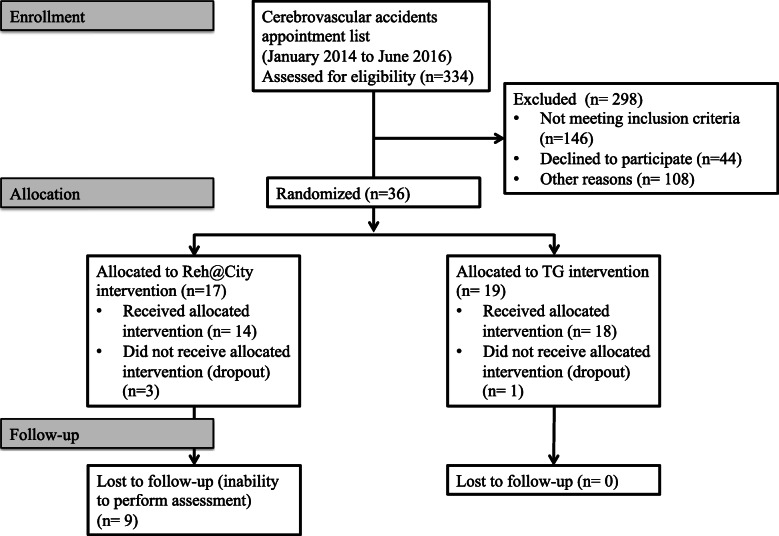
Protocol of the intervention

The sample was selected from a list of 334 stroke patients enrolled in the cerebrovascular accidents appointment list from the Physical Medicine and Rehabilitation department of the Madeira Health Service (Portugal). They were contacted by phone by one of the researchers, and 44 declined to participate, 146 patients were excluded for not meeting the inclusion criteria and 108 were excluded for other reasons, such as transportation problems or lack of response after three phone calls. Overall, 36 patients were included meeting all inclusion criteria and were allocated to one of the two interventions (TG or Reh@City v2.0), by the two psychologists involved in the data collection. Group allocation was randomized (through a simple randomization method using a web-based application that generates a random allocation sequence) among the different rehabilitation units working under the Physical Medicine and Rehabilitation department (Fig. 1).

### Intervention protocol

The study started in January 2017 and stopped in December 2018, since the authors defined the maximum of 2 years for data collection. In total, 19 participants were allocated to the TG group (one dropped out) and 17 allocated to the Reh@City v2.0 group (three dropped out). All patients went through neuropsychological assessment pre and post-intervention and at 2 months follow-up. Each one of the assessment times had an approximate duration of 90 min.

The intervention personalization was done through the characterization of each participant with the MoCA [63, 64] assessment results: the *Attention* parameter was defined from MoCA’s attention component score [0–6]; the delayed recall and orientation scores [0–11] was used to parameterize *memory; executive functions* was parameterized through the sum of the visuospatial, executive, and abstraction MoCA subscores [0–7]; MoCA’s naming and the language scores [0–6] was used to parameterize *language;* and the total score [0–30] was used to parameterize the overall *difficulty.*

Two psychologists performed all the assessments. The same two psychologists and one occupational therapist supervised the interventions sessions. Accordingly, this study was not blind.

### Interventions description

#### Paper-and-pencil intervention: the task generator

**Fig. 2:**
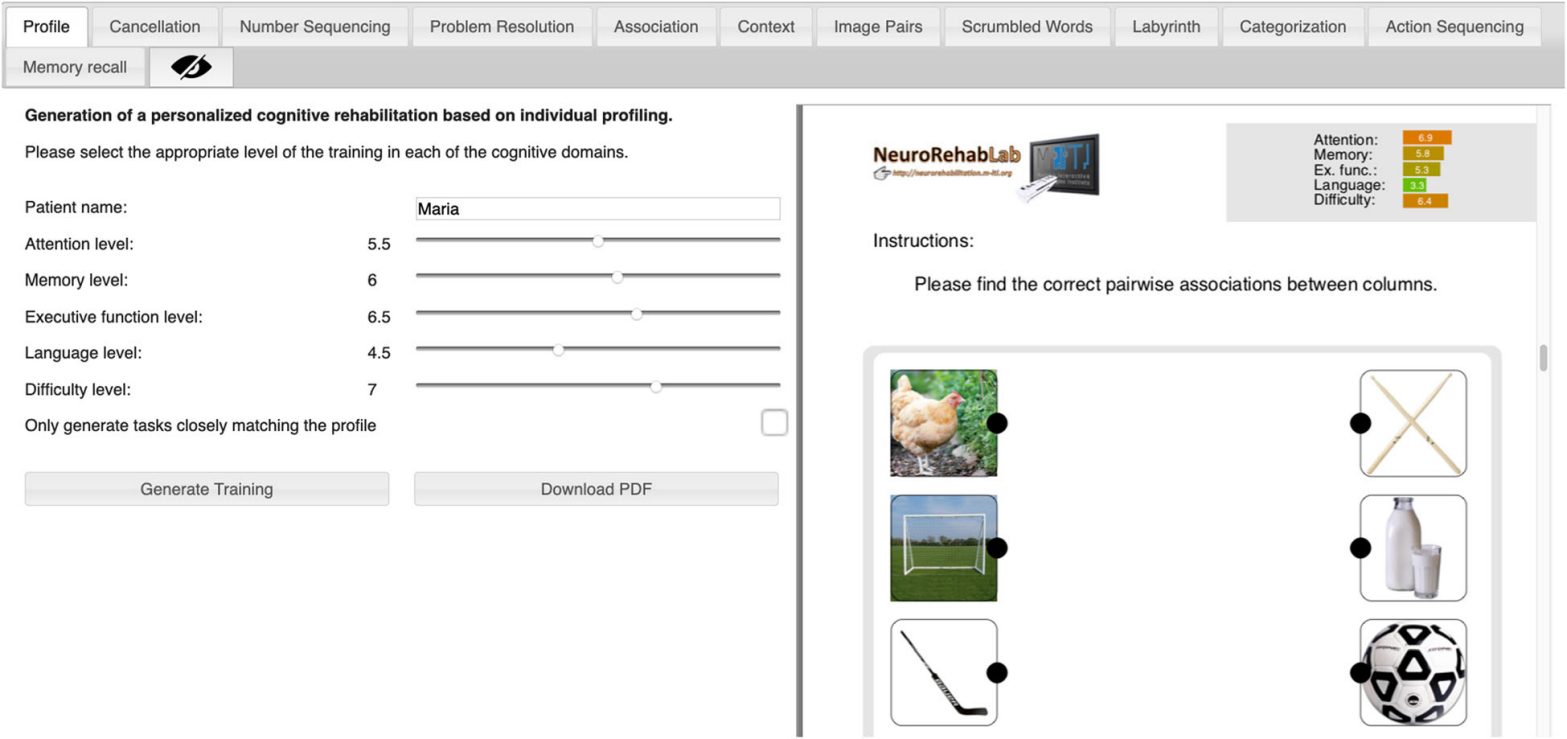
TG training personalization parameters (on the left) and Association task generation example (on the right)

The TG is a free and worldwide accessible tool that is able to generate personalized paper-and-pencil cognitive rehabilitation programs in PDF format, composed by a set of 11 tasks gathered from clinical settings and parameterized through a participatory process with rehabilitation experts [24]: Cancellation; Numeric Sequences; Problem Resolution; Association; Comprehension of Contexts; Image Pairs; Word Search; Mazes; Categorization; Action Sequencing; and Memory of Stories (see Annex 1 for an example). After the characterization of each participant, the MoCA assessment data was normalized on a 1 to 10 scale, and a full training program was generated (Fig. 2). In the TG intervention, participants were instructed to use the arm they would feel more comfortable.

#### VR-based intervention: the Reh@City v2.0

Our VR-based intervention consisted of the same TG paper-and-pencil tasks contextualized in different locations of a virtual city with streets, sidewalks, buildings, shops, and parks – the Reh@City v2.0 [67] (Table 1).

**Table 1:** TG paper-and-pencil tasks correspondence with Reh@City v2.0 VR tasks

Task Generator	Reh@City v2.0
Cancellation - Find a target stimulus in a pool of distractors.	Buy/collect items at the supermarket, pharmacy, and post-office.
Numeric Sequences - A numeric sequence is given and the subject has to come up with the missing numbers.	Find bank code.
Problem Resolution - Two types of problems are presented, numeric calculations or calculations based on textual descriptions of daily activities.	Choose the correct supermarket invoice.
Association - A number of randomized pairs of items need to be paired correctly.	Cards game at the park.
Comprehension of Contexts - Some images are given with a number of descriptions. Correct descriptions need to be identified.	Not applicable.
Image Pairs - A number of pairs of images to be memorized is presented and have to be recalled after 30 min.	Cards game at the park.
Word Search - A number of words can be found up, down, forward, or diagonally in a pool of randomized letters.	Not applicable.
Mazes - Finding the way out of a labyrinth.	Find the best route to the next destination in the virtual city.
Categorization - Grouping items into their underlying categories. The categories have to be guessed from the items.	Select a category of items in the clothing shop.
Action Sequencing - A list of randomized steps needed for the execution of several activities of daily living is presented.	Organize the steps for an action in the home kitchen, living room or bathroom.
Memory of Stories - Recalling information about a read story or a picture by answering questions about it.	Memorizing verbal information from a newspaper at the kiosk for a later “true or false” recall.

**Fig. 3:**
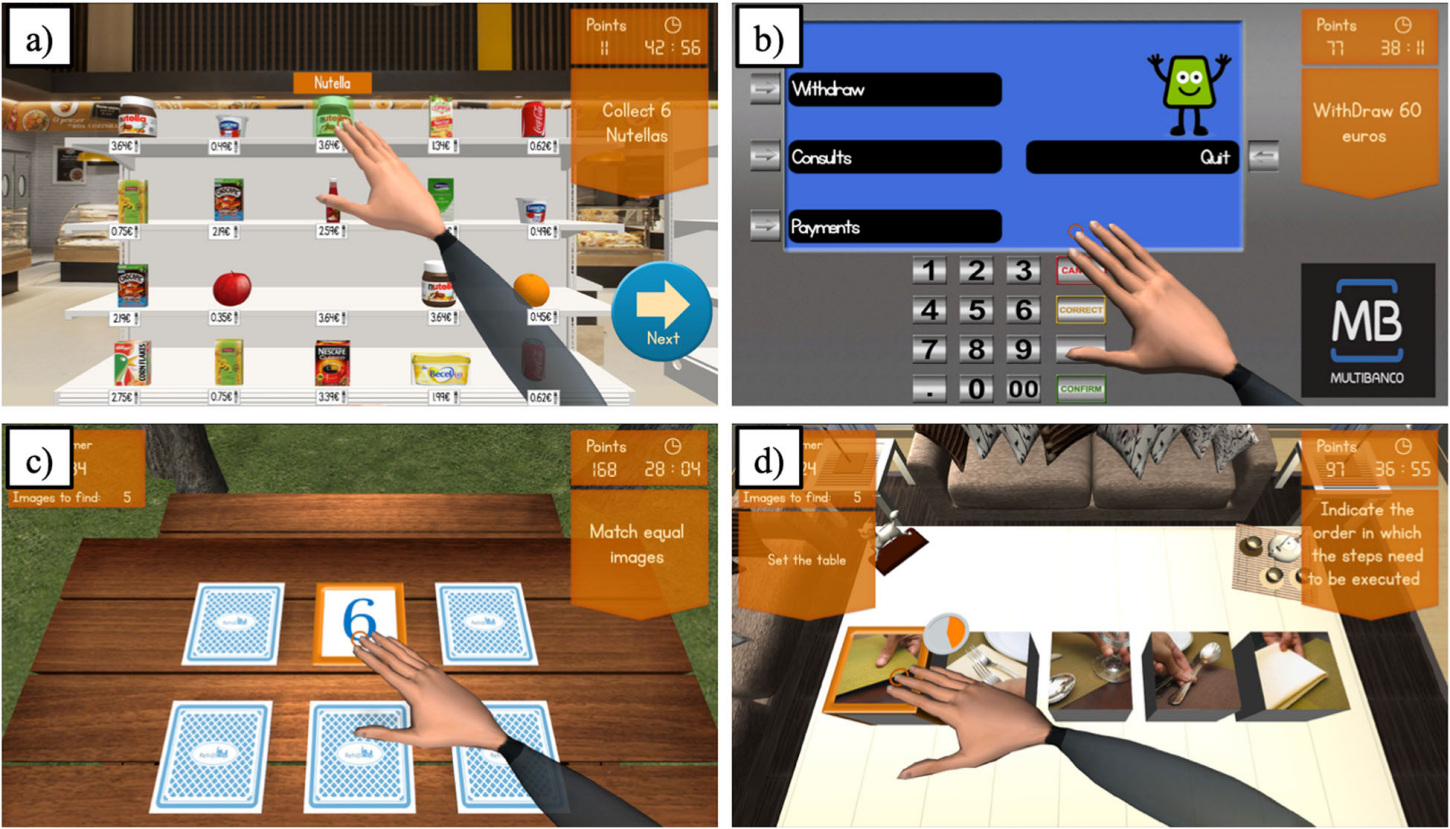
Reh@City v2.0 task examples: **a** buying food in the supermarket; **b** making payments at the bank ATM; **c** playing a cards game at the park and; **d** setting the table at home

Reh@City v2.0 provides a more ecological training experience since patients are required to solve cognitive tasks through familiar ADL’s in a variety of commonplaces: for instance buy food in a supermarket (Fig. 3a); pick up a package in the post office; pay the electricity at the bank ATM (Fig. 3b); buy pain killers in the pharmacy; collect shirts in the clothing shop; play a game in the park (Fig. 3c); read the newspaper in the kiosk and set the table at home (Fig. 3d). These places display billboards and real products of actual spaces and trademarks commonly found in Portugal to help the patient relate the VR tasks to the real world. In addition, consistent with the actual simulated ADLs and to increase the ecological validity of the training, patients were also required to use their paretic arm to solve the tasks.

**Fig. 4:**
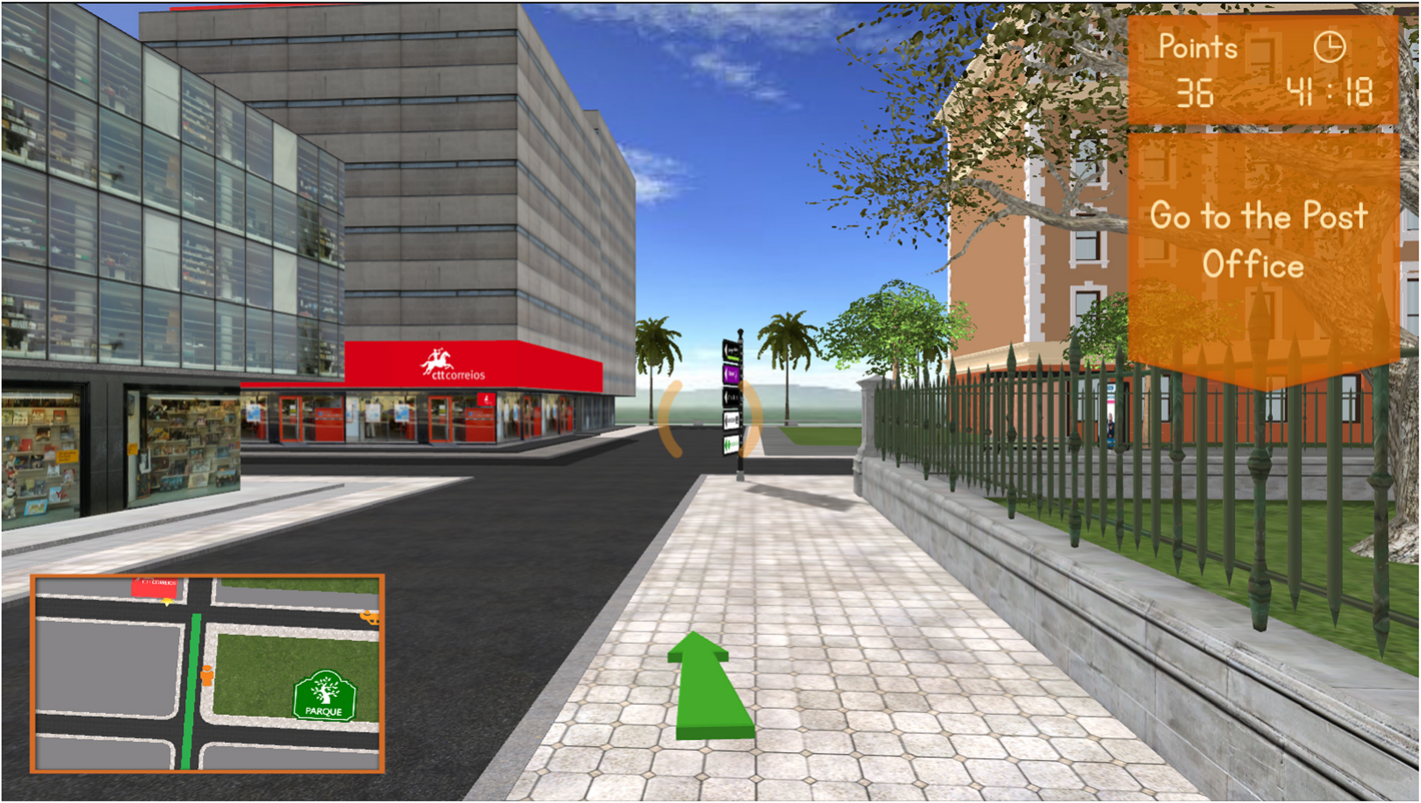
Reh@city v2.0 three-dimensional street view. Users are given goal instructions supported with a mini-map indicating the optimal path and a street arrow. Time and point counters are used to provide feedback on performance

Because we were generally dealing with people of older age and low computer literacy, the interaction with the virtual environment was simplified and city was designed to have only square or rectangular building blocks and perpendicular street intersections, as well as simplified simulated environments. This simplified arrangement also allowed a more precise control of difficulty parameterization (Fig. 4).

VR simulations demand a trade-off of less realism to allow more real-time interactivity and difficulty parameterization. Notwithstanding this simplification, Reh@City has many elements attesting for its ecological validity. In terms of verisimilitude, the tasks simulate everyday life activities, although the performance of the virtual task is facilitated; the real environment and objects are minimally simulated and correspond to familiar locations and trademarks existing in Portugal; the user’s upper limb is partially represented; the specific situations in which actions take place are minimally modeled (for instance, action sequencing of baking a cake happens in a kitchen environment and reading a newspaper article happens in kiosk); it combines 2D, and 3D stimuli and; the navigation in the city is consistent with the point of view of the user. Concerning veridicality, in a previous study with Reh@City v1.0 [29], we have found a transfer of the virtual training to real-world functioning through significant improvements on a functional scale.

#### TG and Reh@City personalization and adaptation

The TG and the Reh@City v2.0 are two content equivalent rehabilitation tools developed under the same personalization algorithm [23]:

$$ DV= intercept+\mathrm{C}1\ast \mathrm{IV}1+\mathrm{C}2\ast \mathrm{IV}2+\dots +\mathrm{C}\mathrm{i}\ast \mathrm{IV}\mathrm{i} $$

where Ci indicates the contribution of each Independent Variable (IV) (task parameters) to the Dependent Variable (DV) (memory, executive functions, attention, language, and difficulty).

Concerning the session-to-session adaptation, when the patient finishes the first set of tasks, a score is computed using a 0 to 100% scale. Consistent with previous adaptive systems for stroke rehabilitation [68], if the mean performance is higher than 70%, the difficulty is increased by 0.5 in the next set of tasks, and if performance is from 0 to 50%, the difficulty parameter is reduced by 0.5.

In both personalization and adaptation, difficulty is one of the DV we have obtained from our algorithm [23], it is related with general cognitive demand.

### Experimental setup

#### Task generator (TG)

The TG is an online application, accessible at neurorehabilitation.m-iti.org/TaskGenerator, and does not require to be installed on the computer. Through this tool, clinicians defined appropriate parameters of training for memory, attention, executive functions, language, and difficulty (obtained from the MoCA screening tool), and it automatically generated a set of the 11 personalized cognitive training tasks. The only required software was a PDF reader to open the downloaded paper-and-pencil tasks. After printed, the tasks were solved on a table with a pencil having the user seated.

#### Reh@City v2.0

**Fig. 5:**
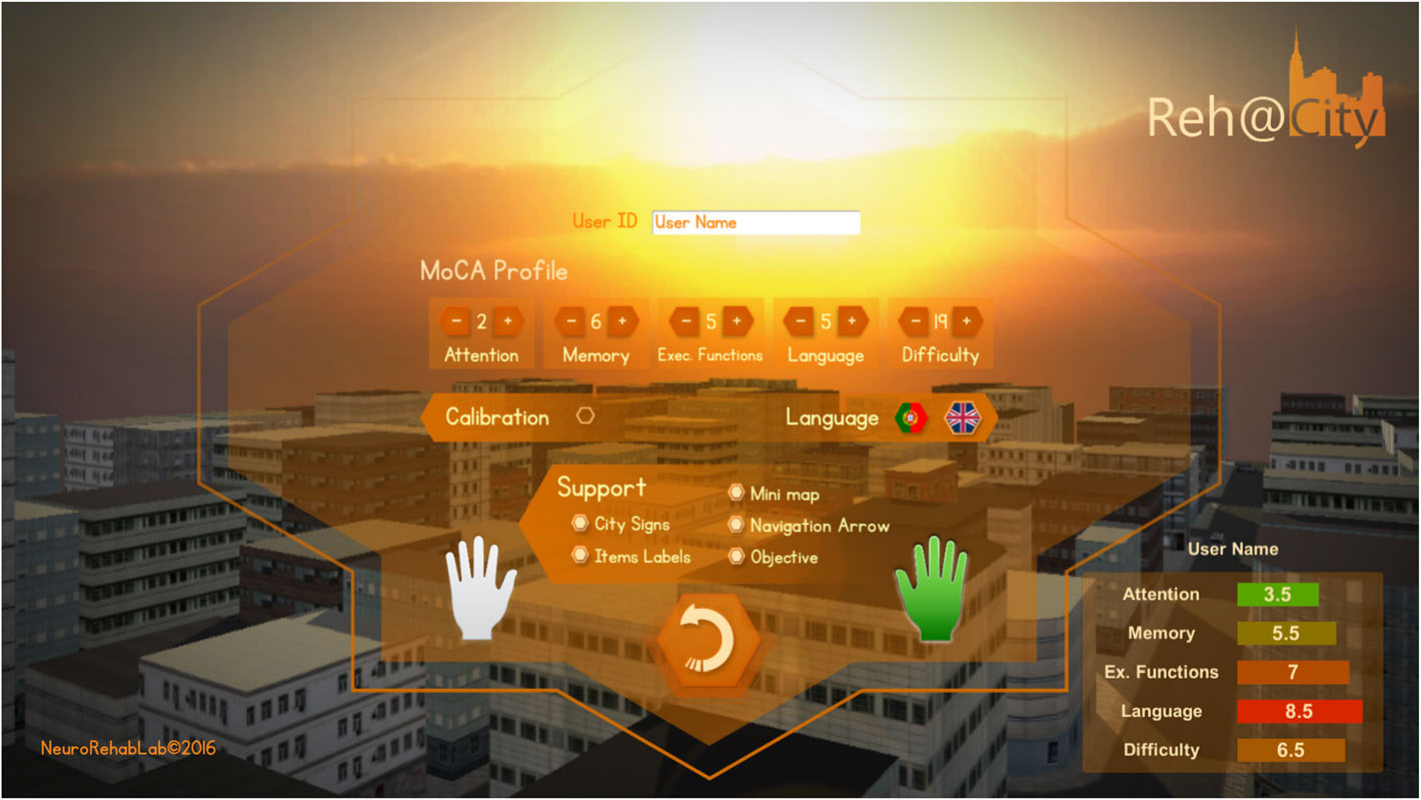
Reh@City v2.0 training personalization parameters according to MoCA total and subdomains score

Reh@City v2.0 was installed on a PC (OS: Windows 7, CPU: Intel core 2 duo E8235 at 2.80 GHz, RAM: 4 Gb, Graphics: ATI mobility Radeon HD 2600 XT). Reh@City is a cognitive rehabilitation tool, that allow clinicians to personalize parameters of training for memory, attention, executive functions, language, and difficulty according to the MoCA total and subdomains score (Fig. 5) and it enables that through upper limb movements.

**Fig. 6:**
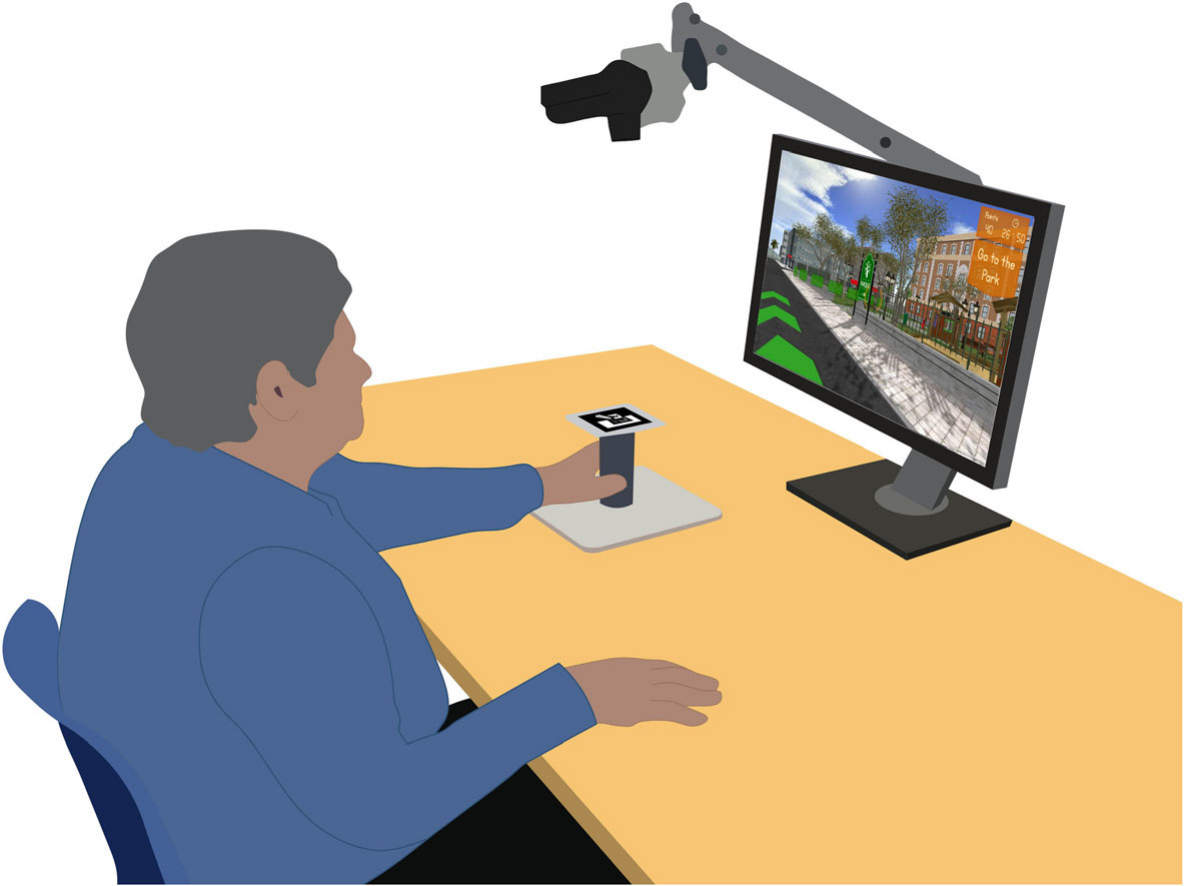
Reh@City v2.0 experimental setup. The user faces an LCD monitor and moves a handle on the surface of the table with his/her paretic arm to interact with the virtual content

Given the potential benefits reported in the literature of combining cognitive and motor rehabilitation through VR [30–32], Reh@City v2.0 implies the use of the paretic arm to solve its cognitive training tasks. The user worked on a tabletop, facing an LCD monitor (24″) and moved a customized handle with a tracking pattern on the surface of the table with his/her paretic arm (Fig. 6). 2D upper limb reaching movements were captured through a camera-based Augmented Reality (AR) pattern tracking software (AnTS) [69] connected to a PlayStation Eye camera (Sony Computer Entertainment Inc., Tokyo, Japan). For adapting the interaction to individual users, the Reh@City v2.0 implemented a built-in calibration function that normalizes the motor effort required in the task to the active range of movement of the user. The movements of the user are then mapped onto the movements of a virtual arm (in indoor tasks) or as movement directions (during outdoor navigation) in the Reh@City v2.0 environment.

### Outcome measures

#### Primary outcome measures: general cognitive functioning, attention, memory, executive functions and language

As primary outcome measures we used the MoCA [63, 64] as a general cognitive functioning measure, which has been reported to have a good sensitivity and specificity in screening for cognitive impairment after stroke [70]. In addition, the decline in MoCA scores (reduction ≥2 points) was found to be associated with the decline in neuropsychological diagnosis transitional status on a sample of 275 stroke patients [71].

Moreover, we selected specific attention, memory, executive functions, and language assessments, which are the domains targeted by both TG and Reh@City v2.0. To assess attention we used the Trail Making Test A and B (TMT A and B) [72, 73], a very popular neuropsychological test that provides information on visual search, visual scanning, selective and divided attention, processing speed, mental flexibility, and also executive functioning. In part A, circles numbered from 1 to 25 need to be connected in numerical order. In part B, numbers from 1 to 13 and letters from A to L need to be connected alternating numbers and letters in ascending order. The memory assessment was performed with the Verbal Paired Associates from the Wechsler Memory Scale-III (WMS-III) [74]. To assess executive functions, namely working memory and processing speed, we used the Digit Span (forward and backward recall conditions) also from the WMS-III, and the Symbol Search and the Digit Symbol Coding (codification and incidental learning pairing conditions) from the Wechsler Adult Intelligence Scale III (WAIS) [75]. Finally, we assessed language through the Vocabulary from WAIS-III, which provides information about verbal comprehension.

#### Secondary outcome measure: self-perceived impact of cognitive functioning problems

As secondary outcome measure, we assessed the perceived impact of persisting problems with cognition, as assessed by the Patient-Reported Evaluation of Cognitive State (PRECiS) [76, 77], which includes 27 core items asking respondents about the impact of cognition on four conceptual dimensions: everyday life skills, family and life, mood and sense of self.

### Statistical analysis

All statistical analyses were performed using SPSS software (version 20, SPSS Inc., Chicago IL, USA). As a criterion for significance, we used a α of .050. With Bonferroni correction the α was .002, as such significant differences with corrected *p*-values are also mentioned. Normality of data was assessed with the Kolmogorov-Smirnov (KS) test. As some data were not normally distributed, nonparametric tests were used to evaluate the inter-group and intra-group differences. The Wilcoxon signed-rank test (W) was used to analyze the within group changes over time, while the two-tailed Mann-Whitney (MW) test was used to compare the between-group differences from baseline to the end of the study. Demographic differences between groups were measured with the Mann Whitney test (MW). Effect sizes (*r*) were computed as *Z*/√*N* on the pairwise comparisons. The criteria for interpretation of the effect was 0.1 = small, 0.3 = medium, and 0.5 = large.

## Results

### Sample description

The sample consisted of thirty-two patients with stroke randomly distributed in two groups. The Reh@City v2.0 group comprised fourteen (5 male, 9 female) adult (*M* = 59.1 years old, *SD* = 11.8) patients with stroke (11 right hemisphere, 3 left hemisphere; 12 ischemic, 2 hemorrhagic), with an average of 45.9 ± 43.6 months post- stroke and a mean of 8 ± 5.3 years of schooling. The TG group comprised eighteen (11 male, 7 female) adult (*M* = 65 years old, *SD* = 6.2) patients with stroke (9 right hemisphere, 6 left hemisphere, 3 not specified; 14 ischemic, 3 hemorrhagic, 1 not specified), with an average of 21.3 ± 12.9 months post-stroke and a mean of 5.5 ± 3.2 years of schooling. The Mann-Whitney test revealed no differences between groups in the demographic characteristics and in all baseline outcome measures (Table 2).

**Table 2:** Demographic characteristics (presented as Means ± SD’s) of the two groups and differences between groups measured by the Mann-Whitney test (MW)

	Reh@City v2.0 (*N* = 14)	Task Generator (*N* = 18)	MW	*p*
Age (years)	59.14 ± 11.81	65.00 ± 6.20	83.500	.106
Gender (M/F)	5/9	11/7	94.000	.161
Schooling (years)	8.00 ± 5.32	5.50 ± 3.15	100.500	.276
Stroke type (I/H/NS)	12/2/0	14/3/1	115.000	.538
Side of lesion (R/L/NS)	11/3/0	9/6/3	85.500	.072
Time post-stroke (months)	45.93 ± 43.56	21.33 ± 12.88	89.500	.164

Sex: F, female; M, male; Schooling is presented in years; Type of stroke: I, ischemic; H, hemorrhagic; NS, not specified; Side of lesion: L, left; R, right; NS, not specified; Time post-stroke is presented in months

According to the Kolmogorov-Smirnov (KS) test, data were normally distributed in both groups for age (KS_Reh@City_ = .189, *p* = .190; KS_TG_ = .182, *p* = .118) and time post-stroke (KS_Reh@City_ = .211, *p* = .091; KS_TG_ = .187, *p* = .095). Data were not normally distributed for gender (KS_Reh@City_ = .407, *p* = <.001; KS_TG_ = .392, *p* = <.001), type of stroke (KS_Reh@City_ = .510, *p* = <.001; KS_TG_ = .463, *p* = <.001), the side of lesion (KS_Reh@City_ = .510, *p* = <.001; KS_TG_ = .463, *p* = <.001), and the number of years of schooling (KS_Reh@City_ = .345, *p* = < .001; KS_TG_ = .405, *p* = < .001).

### Primary outcome measures

#### MoCA - general cognitive functioning

We analyzed the global cognitive functioning, as assessed by the MoCA, of the two groups in the pre and post intervention assessments, and follow-up for the Reh@City v2.0 and TG groups (Table 3).

**Table 3:** MoCA scores (presented as Medians and IQR) pre and post intervention and follow-up highlighted for within-groups significant differences and marked with an asterisk for between-groups significant differences

	Reh@City v2.0			Task Generator		
	Pre	Post	FU	Pre	Post	FU
Total	23 (19.8–26)	25 (23–27.3)*	28 (22.5–28.5)	21 (18.8–24.3)	21 (16.8–23.3)	23 (19.8–25.3)
Visuo-Executive	3.5 (2.8–4)	**4 (3–5)**	4 (4–5)	3.5 (2–4)	4 (3–4)	4 (2.8–5)
Naming	3 (2–3)	3 (2.8–3)	3 (1.5–3)	2.5 (2–3)	2 (1–3)	3 (2–3)
Attention	4 (2.8–5.3)	**5.5 (3–6)**	5 (3–6)	4 (2–5.3)	4 (2.8–5)	4 (3–5.2)
Language	2 (1.8–3)	2 (2–3)	3 (2–3)	2 (2–3)	2 (1–2)	2 (1–2)
Abstraction	2 (1–2)	2 (1–2)	2 (1–2)	1 (1–2)	2 (0–2)	1 (1–2)
Memory	3 (1–3.5)	3 (2–4)	4 (2.5–5)	2 (0–3.3)	.50 (0–2)	2.50 (1.8–3.2)
Orientation	6 (6–6)	6 (6–6)	6 (6–6)	6 (5–6)	**6 (6–6)**	6 (6–6)

A Wilcoxon test for within-groups differences revealed that only the Reh@City v2.0 group presented significant statistical improvements between pre and post assessment times in MoCA [Pre: Mdn = 23, IQR = 19.8–26; Post: Mdn = 25, IQR = 23–27.3 (*W*_(14)_ = 64.00, *Z* = − 2.777, *p* = .005, *r* = .74)]. In the subdomains analysis, we found significant improvements in visuospatial ability and executive functioning [Pre: Mdn = 3.5, IQR = 2.8–4; Post: Mdn = 4, IQR = 3–5 (*W*_(14)_ = 41.00, *Z* = − 2.310, *p* = .021, *r* = .62)] and attention [Pre: Mdn = 4, IQR = 2.8–5.3; Post: Mdn = 5.5, IQR = 3–6 (*W*_(14)_ = 28.00, *Z* = − 2.460, *p* = .014, *r* = .66)].

Concerning the TG group, the only significant change was in the MoCA orientation subdomain [Pre: Mdn = 6, IQR = 5–6; Post: Mdn = 6, IQR = 6–6 (*W*_(18)_ = 15.00, *Z* = − 2.121, *p* = .034, *r* = .57)].

A Mann-Whitney test indicated that the Reh@City v2.0 group improved significantly more than the TG group, in terms of general cognitive functioning, as assessed by the MoCA, from baseline to post-intervention [Reh@City v2.0: Mdn = 2, IQR = 0–3; TG: Mdn = − 1.5, IQR = − 3.25–2 (*U* = 65.00, *Z* = − 2.334, *p* = .020, *r* = .41)].

#### TMT A & B - attention

We computed the TMT A and TMT B performance for the two groups, in terms of errors and completion time, pre, post-intervention and follow-up (Table 4). Only the TG group showed a significant improvement in the reduction of time to completion of the TMT A test pre to post-intervention [Pre: Mdn = 84, IQR = 59.5–114.3; Post: Mdn = 72, IQR = 58.8–99.5 (*W*_(18)_ = 18.00, *Z* = − 2.588, *p* = .010, *r* = .61)].

**Table 4:** TMT A and B; WMS-III Verbal Paired Associates (VPA); and WAIS-III Digit Symbol Coding (DSC), Symbol Search, Digit Span and Vocabulary scores (presented as Medians and IQR) pre and post intervention and follow-up highlighted for within-groups significant differences

		Reh@City v2.0			Task Generator		
		Pre	Post	FU	Pre	Post	FU
TMTA	time	72.5 (49.5–97.5)	65 (51–86.3)	70 (30.5–84)	84 (59.5–114.3)	**72 (58.8–99.5)**	76.5 (59.3–114.3)
	errors	0 (0–0)	0 (0–0)	0 (0–0)	0 (0–0)	0 (0–0)	0 (0–.50)
TMTB	time	195 (130.8–360)	200 (135.5–241)	190 (61.5–360)	209.5 (123.3–256.5)	236 (152–360)	202 (112.3–360)
	errors	0 (0–3)	0.5 (0–1.25)	1 (0–4.5)	3 (1.5–6)	3 (1–4.5)	2.5 (0–3.8)
WMSIII	VPA learning	2 (.75–4)	1.5 (1–4)	5 (1–6)	1 (0–2)	1 (.75–2.3)	1.50 (8–4)
	VPA retention	75 (0–100)	100 (74.1–100)	83.3 (25–93.8)	.00 (0–56.3)	82.9 (26.5–100)	82.9 (37.5–100)
	VPA recognition	24 (21.8–24)	24 (24–24)	24 (24–24)	23 (19.8–24)	24 (21–24)	24 (23.8–24)
WAISIII	DSC codification	28.5 (23.5–36.8)	33 (26.8–47)	33 (19–50)	21.5 (11.8–33)	26.5 (18.8–38.3)	27 (16.8–34.3)
	DSC incidental learning	3 (.0–10.5)	6.5 (4–11.5)	10 (3–16)	4 (1.5–8.8)	6 (.8–10.5)	8 (2–14)
	Symbol Search	13.5 (9.8–20.5)	17.5 (10.3–24)	17 (10–25.5)	12 (7.8–13.5)	14 (10–16.5)	15 (9–20.3)
	Digit Span	11 (10–13)	10 (8.8–13)	11 (10.5–13.5)	10 (8–11)	10.5 (8.8–12)	10 (9.5–13.3)
	Vocabulary	29 (21–34)	25.5 (12.8–30.3)	22 (13.5–40)	19.5 (13–28.5)	20.5 (12.8–30.3)	**24.5 (16.5–30.3)**

#### WMS-III verbal paired associates - memory

Table 4 describes the Verbal Paired Associates test performance for the two groups pre, post-intervention and follow-up. In this learning and memory test, we found significant improvements within the Reh@City v2.0 group for the retention [Pre: Mdn = 75, IQR = 0–100; Post: Mdn = 100, IQR = 74.1–100 (*W*_(14)_ = 36.00, *Z* = − 2.524, *p* = .012, *r* = .67)] and recognition [Pre: Mdn = 24, IQR = 21.8–24; Post: Mdn = 24, IQR = 24–24 (*W*_(14)_ = 21.00, *Z* = − 2.214, *p* = .027, *r* = 59)] scores post-intervention. In the TG group improvements were only significant for the retention score in both post-intervention [Pre: Mdn = 0, IQR = 0–56.3; Post: Mdn = 82.9, IQR = 26.5–100 (*W*_(18)_ = 118.00, *Z* = − 2.602, *p* = .009, *r* = .61)] and follow-up [Pre: Mdn = 0, IQR = 0–56.3; FU: Mdn = 82.9, IQR = 37.5–100 (*W*_(18)_ = 95.00, *Z* = − 2.776, *p* = .006, *r* = .65)].

WAIS-III Digit Symbol Coding, Symbol Search and Digit Span - Executive Functions.

Table 4 describes the executive functioning outcome measures for the two groups pre, post-intervention and follow-up. The Reh@City v2.0 group showed improvements in the Digit Symbol Coding codification task post-intervention [Pre: Mdn = 28.5, IQR = 23.5–36.8; Post: Mdn = 33, IQR = 26.8–47 (*W*_(14)_ = 87.00, *Z* = − 2.171, *p* = .030, *r* = .58)]. The TG group had significant improvements in the Symbol Search at follow-up [Pre: Mdn = 12, IQR = 7.8–13.5; FU: Mdn = 15, IQR = 9–20.3 (*W*_(18)_ = 101.00, *Z* = − 2.340, *p* = .019, *r* = .55)].

#### WAIS-III vocabulary - language

The analysis of the language outcome measure for the two groups pre, post-intervention and follow-up revealed that only the TG group showed improvements in the Vocabulary assessment at follow-up [Pre: Mdn = 19.5, IQR = 13–28.5; FU: Mdn = 24.5, IQR = 16.5–30.3 (*W*_(18)_ = 166.00, *Z* = − 3.514, *p* < .001, *r* = .83)] (Table 4). This difference was also significant with Bonferroni correction (*p* = .002).

### Secondary outcome measure

#### Patient-reported evaluation of cognitive state

When analyzing the answers of the two groups pre, post-intervention and follow-up to the PRECiS questionnaire, only the Reh@City v2.0 group revealed a significant self-perceived decrease in the stroke cognitive deficits impact post intervention [Pre: Mdn = 13.5, IQR = 7–23.8; Post: Mdn = 12, IQR = 3.8–21.3 (*W*_(14)_ = 13.00, *Z* = −2.041, *p* = .041, *r* = .55)] (Table 5).

**Table 5:** PRECiS score (presented as Medians and IQR) pre and post intervention and follow-up highlighted for within-groups significant differences

	Reh@City v2.0			Task Generator		
	Pre	Post	FU	Pre	Post	FU
PRECiS	13.5 (7–23.8)	**12 (3.8–21.3)**	13 (0–24.5)	28.5 (6–47)	18.5 (8.5–44.8)	13.5 (5.5–30.3)

## Discussion

In the last years there has been significant growth in the evidence for post-stroke cognitive rehabilitation [12, 13], with a number of studies proposing ecologically valid VR-based simulations of ADL’s as the most promising training solutions [26, 27]. Notwithstanding the advantages of VR, there are several areas that require further progress. One is the need to bridge widely accepted paper-and-pencil methodologies with VR-based ADL’s simulations. In the field of cognitive assessment, some studies have compared VR cognitive assessment tools with their paper-and-pencil original versions [78–80]. Existing studies about VR simulations of cities showed convergent validity between some measures of the performance in simulated virtual cities and clinical neuropsychological tests of variable strength, which ranges from moderate for attention [37], good to excellent for executive functioning [37], and excellent for general cognitive condition [28]. Poor to moderate correlations have also been reported between navigation in a real and a virtual city [34]. If we look at cognitive training, to the best of our knowledge, there is not much work that we can compare with our study. In terms of VR-based interventions, Gamito and colleagues used a virtual city with everyday life simulations with stroke patients and have found improvements in attention and memory [36]. Related with Task Generator paper-and-pencil training, there are the Guttman Neuropersonal Trainer [19] and the Cogweb [20] that are customizable and have already some evidence about its efficacy in cognitive training.

Another limitation about VR-based cognitive rehabilitation tools is that, an important number of the developed systems have not been field tested [38], have only gone through studies with a small number of participants [29, 36] and/or with healthy control groups [78]. Additionally, none of these VR-based ADL’s simulations are compared with widely used and clinically accepted paper-and-pencil tasks, being always compared with non-equivalent interventions as OT [81]. Our RCT, is the first to implement an adaptive paper-and-pencil training and compare it with a content equivalent VR-based ADL’s simulation, by using the same tasks, personalization and difficulty adaptation framework within a longitudinal clinical intervention.

TG and the Reh@City v2.0 groups differed in the motor demand of the training; while the TG group used a pencil with the healthy arm to solve the tasks, the Reh@City v2.0 group used an adapted handle with the affected arm, performing 2D upper limb reaching movements to interact with the tasks. Assuming the interdependence between the recovery processes [32], we may have provided a more effective rehabilitation to the Reh@City v2.0 group in both cognitive and motor domains; however, motor recovery is not an outcome in this study and, consequently, was not assessed. The Reh@City v2.0 main goal is to be an ecologically valid cognitive training tool, and the use and virtual representation of the paretic arm to solve the ADL’s simulations was implemented to increase ecological validity. As such, the difference in motor demand is intrinsic to the research hypothesis. If we asked the TG group to use the paretic arm, we would be harming the resolution of the paper-and-pencil tasks, thereby, Reh@City v 2.0 and TG interventions could not be fairly compared.

### Primary outcome measures

The Reh@City v2.0 group improved in the MoCA general cognitive functioning and in its attention, visuospatial ability and executive functioning subdomains, with large effect sizes. The TG group improved in the MoCA orientation domain, also with a large effect size. In a between groups comparison, the Reh@City v2.0 group had a higher impact in the general cognitive functioning comparatively to the TG group, with a medium effect size. Wong and colleagues (2017) determined that the MoCA score change associated with a change of health in general, in a sample of 175 aneurysmal subarachnoid hemorrhage patients, was of two points [82]. The generalization of this finding to our study is consistent with this between-groups difference, since the Reh@City v2.0 group improves its median score from 23 to 25 points in the post-intervention, while the TG maintains the median score of 21 points. At the follow-up, Reh@City v2.0 improves its median score to 28 points, while the TG has a more discrete improvement to 23 points. Meaning that the Reh@City v2.0 group was the only to present Minimum Clinically Important Differences (MCID) from baseline to end, and both from end to follow-up.

Despite the fact that both Reh@City v2.0 and TG are content equivalent interventions that follow the same personalization guidelines and difficulty progression rules, the superiority of the impact of the VR-based intervention was expected since it reunites a number of promising features that distinguishes it from the paper-and-pencil intervention: 1) it is an ecologically valid ADL’s simulations; 2) stimulus are everyday-life brands and products; 3) it has game elements so participants are rewarded for successful performance in real time and; 4) it provides immediate feedback enabling higher success in accomplishing the tasks. Although with a different primary outcome measure, these results are coherent with a previous Reh@City v1.0 study, where the Reh@City v1.0 group improved in the Addenbrooke Cognitive Examination [83] general cognitive functioning, attention, memory and visuospatial abilities and was superior between groups in general cognitive functioning, attention and fluency (where the control group had a significant decrease) [29]. Reh@City v1.0 was an initial prototype with four commonly frequented places of daily life (Pharmacy, Bank, Supermarket, and Post-office) [28]. The upgrade to Reh@City v2.0 included: an increase of the ecological validity through the improvement in the overall visual realism of the city and existing tasks, and the interaction through the paretic arm using an adapted handle; the implementation of dynamic difficulty adaptation based on the framework we developed for TG tasks [23]; and the increase of the number of cognitive training tasks and locations (Magazine Kiosk, Home, Park, and Fashion Store) [67].

Also, the Reh@City v2.0 intervention group had a significant impact, and with large effect sizes, in verbal memory (as assessed by the retention and recognition from the Verbal Paired Associates test - WMS-III), and processing speed (as assessed by the Digit Symbol Coding task - WAIS-III), which is superior to what we have found in the Reh@City v1.0 study, where we only had improvements in the executive functioning measure (the Picture Arrangement test from the WAIS-III) and the control group improved in the reduction of the number of errors in the TMT B, a processing speed and selective and divided attention measure [29]. This superiority of the Reh@City v2.0 concerning verbal memory and processing speed was expected since it has an increased number of cognitive training tasks and locations in comparison to Reh@City v1.0, that did not even had verbal memory training.

The TG group improved in verbal memory (as assessed by the retention from the Verbal Paired Associates test - WMS-III) and processing speed (as assessed by the TMT A task execution time) subdomains, with large effect sizes. At follow-up, participants who underwent the TG intervention maintained the verbal memory benefits with new improvements in the sustained attention and processing speed (as assessed by the Symbol Search task – WAIS-III) and language (as assessed by the Vocabulary – WAIS-III) domains, also with large effect sizes. These findings may be related to the fact that the TG offers a more domain-specific training and recent evidence supports that attention, language [14], memory, executive functions and visuospatial and perceptual skills [13] training after stroke is effective.

### Secondary outcome measure

Besides cognition, we assessed the intervention’s impact in the self-perceived cognitive deficits. Only the Reh@City v2.0 group had a significant reduction in the self-perceived cognitive deficits in different aspects of their everyday life (everyday life skills, family and life, mood and sense of self), measured by the PRECiS questionnaire with a large effect size. This finding is in line with existing literature that states that, comprehensive neuropsychological rehabilitation, is effective to reduce functional disability after stroke [14]. In the Reh@City v1.0 study we assessed the intervention’s impact in the multiple domains of health and life with a different outcome measure - the Stroke Impact Scale 3.0 [84]. In this self-report questionnaire, the VR group improved significantly in the physical domain, memory, emotion, social participation and overall recovery while the control group decreased in the physical domain, only improving in memory, mobility and social participation, which supported the superiority of the intervention with the Reh@City v1.0 in comparison to the control group.

### Limitations

Some limitations of our study must be considered when interpreting the results. Concerning the sample, although 42 stroke participants is a small sample it is comparable to previous similar clinical trials [29, 36]. In the perspective of the interventions comparison, Reh@City v2.0 and TG have several intrinsic differences mainly related with the fact that cognitive tasks are presented differently (through VR and paper-and-pencil, respectively). Besides the novelty carried by the use of VR technology in Reh@City v2.0, it requires the subject to use its paretic upper limb while TG does not, as it might be hard or even impossible for a participant to properly use a pencil to solve cognitive tasks with his paretic arm. Although the discrepancies inherent to both interventions do not allow us to know if Reh@City v2.0 impact superiority is related to the use of VR, to the ecological validity of the tasks, or the integration of motor and cognitive training, the main objective of this study was to compare Reh@City v2.0 with a content equivalent intervention that follows the same personalization guidelines and difficulty progression rules (the TG), acknowledging their differences. Also, in the Reh@City v2.0 group, most participants were lost at follow-up. Hence, the comparison between TG and Reh@City v2.0 at this assessment moment should be considered with caution. Furthermore, the intervention was not blind since the same persons performed the assessments and interventions. Also, there might have been learning effects of the cognitive assessment tools, at post-intervention and follow-up assessment times, since only the MoCA had parallel versions for multiple assessments. Yet, even if a learning effect existed, this would apply to both groups, and the comparison would still be valid.

## Conclusions

The results of this one-month longitudinal study showed a positive impact of a rehabilitation training with the Reh@City v2.0, an ecologically valid VR ADL’s simulations, in general cognitive functioning, visuospatial ability and executive functioning, attention, verbal memory and processing speed, generalized for other health and life aspects measured by the self-perceived impact of cognitive deficits scale. This generalization did not happen in the TG group that only revealed similar cognitive impact in the orientation, processing speed and verbal memory domains. The TG intervention sustained impact at follow-up, maintaining processing speed and verbal memory improvements and revealing a new one in language. Finally, by comparing interventions between themselves, we have found Reh@City v2.0 to be superior in general cognitive functioning, visuospatial ability and executive functioning. Only the intervention with TG allowed cognitive gains to last over time. However, these results need to be considered with caution given the dropout at follow-up in the Reh@City v2.0 group.

Overall, our results contribute with new evidence about the impact of ecological validity - using personalization and adaptation in VR simulations of ADL’s and paper-and-pencil tasks - in the rehabilitation of cognitive deficits, which can facilitate the adoption of these innovative tools by health professionals in their daily practice. Nevertheless, there is still a need for further research considering other clinical populations, as well as the implementation of a wider variety of cognitive training tasks.

## Data Availability

The central tendency and dispersion data, as well as the Mann-Whitney and the Wilcoxon tests, from which the conclusions are drawn, are provided in the article. Raw data is available from the corresponding author on reasonable request.

## Abbreviations

ADL’s: Activities of Daily Living
KS: Kolmogorov-Smirnov
MoCA: Montreal Cognitive Assessment
MW: Mann-Whitney
PRECiS: Patient-Reported Evaluation of Cognitive State
RCT: Randomized Controlled Trial
TG: Task Generator
TMT: Trail Making Test
VR: Virtual Reality
W: Wilcoxon
WAIS: Wechsler Adult Intelligence Scale
WMS: Wechsler Memory Scale
